# Higher toenail selenium is associated with increased insulin resistance risk in omnivores, but not in vegetarians

**DOI:** 10.1186/s12986-020-00484-6

**Published:** 2020-08-03

**Authors:** Qiuyun Gu, Xueying Cui, Kun Du, Bian Wang, Wei Cai, Qingya Tang, Xiuhua Shen

**Affiliations:** 1 Department of Nutrition, School of Public Health, Shanghai Jiao Tong University School of Medicine, Shanghai, China; 2 Department of Laboratory Medicine, Xinhua Hospital Affiliated to Shanghai Jiao Tong University School of Medicine, Shanghai, China; 3 Shanghai Key Laboratory of Pediatric Gastroenterology and Nutrition, Xinhua Hospital Affiliated to Shanghai Jiao Tong University School of Medicine, Shanghai, China; 4 Department of Clinical Nutrition, Xinhua Hospital Affiliated to Shanghai Jiao Tong University School of Medicine, Shanghai, 200092 China

**Keywords:** Selenium, Toenail, Insulin resistance

## Abstract

**Background:**

The relationship between selenium (Se) and insulin resistance remains unclear. We aim to explore the association between toenail Se levels and insulin resistance through a cross-sectional study comprising Chinese vegetarians and matched omnivores.

**Methods:**

In this study, we enrolled 220 vegetarians and 220 omnivores matched by age and sex from Shanghai. The inductively coupled plasma mass spectrometry method was used to measure toenail Se levels. Dietary Se intakes were assessed by the 24-h dietary recall method. Blood samples were collected to measure fasting blood glucose level and fasting insulin concentrations. Insulin resistance index (HOMA-IR) and insulin secretion index (HOMA-B) were calculated to evaluate insulin resistance. Multi-linear regression analysis was performed to determine the association between toenail Se levels and insulin resistance, after adjusting for confounders.

**Results:**

The mean ages of vegetarians (76 vegans, 144 lacto-ovo-vegetarians) and omnivores were 35.96 ± 8.73 years and 35.23 ± 8.93 years, respectively. Of these, 180 (81.8%) were female and 40 (18.2%) were male. No association was found between toenail Se levels and insulin resistance in vegetarians. However, the concentration of Se in toenails was positively correlated with fasting insulin levels (*β* = 1.030, 95%*CI*: 0.393 to 1.667) and HOMA-IR (*β* = 0.245, 95%*CI*: 0.098 to 0.392) in omnivores, after multivariate adjustment for age, sex, BMI, alcohol consumption, income, and daily dietary intakes (energy, protein, fat, carbohydrate, and fiber). This positive relationship persisted only in omnivores whose dietary Se intake was above 60 μg/d.

**Conclusions:**

Higher toenail Se levels were associated with increased insulin resistance risk in Chinese omnivores whose dietary Se intake was above 60 μg/d, but not in vegetarians. These findings create awareness on the association of dietary Se intake above 60 μg/d with the risk of insulin resistance.

## Background

Type 2 diabetes mellitus (T2DM) has become a serious global threat to public health with huge economic burden [1]. The International Diabetes Federation estimated that nearly 451 million people living with diabetes were documented worldwide in the year 2017, and these figures are expected to rise to 693 million by the year 2045 [2]. Currently, China has the world’s largest diabetes epidemic, affecting approximately 10.9% of the Chinese adult population [3].

Many previous studies have suggested that vegetarian diet may prevent the development of T2DM and improve insulin resistance [4–6]. A systematic study in Harvard T.H. Chan School of Public Health found a remarkable inverse association between higher adherence to the plant-based dietary pattern and the risk of T2DM (relative risk, 0.77; 95% *CI*, 0.71–0.84) in comparison to poorer adherence [4]. Our previous research revealed that vegetarians have higher insulin sensitivity and lower fasting insulin level in comparison with the matched omnivores [7]. This may be ascribed to the consumption of plant foods rich in antioxidants, vitamins, and minerals and lower consumptions of red and processed meat [4].

Research evidence shows that the intake of Se in vegans did not reach the Nordic Nutrition Recommendations, suggesting a high risk of Se inadequacy in vegans [8–10]. Selenium (Se) is a key component of glutathione peroxidase which suppresses free radicals and lipid peroxides-induced cell damage [11]. Because of the antioxidant properties of selenoproteins and because selenate mimics insulin activity, Se was expected to prevent T2DM [12]. A study based on data from two large cohort studies in the U.S: the Nurses’ Health Study and the Health Professionals Follow-Up Study, found that higher toenail Se level correlated with lower risk for T2DM [13]. However, a recent meta-analysis of experimental and non-experimental studies showed that Se might increase the risk of T2DM across a wide range of exposure levels [14]. Presently, the role of Se in diabetes is not well defined.

Findings on the association between Se and diabetes have been largely inconsistent. Insulin resistance is not only a hallmark but also a pathophysiological factor in diabetes [15]. A few studies have examined the association between Se levels and insulin resistance, but the research findings from such studies are contradictory [16, 17]. Furthermore, no research has evaluated the relationship between Se status and insulin resistance in vegetarians and omnivores.

On the basis of our previous findings, we aim to investigate the association between toenail Se levels and insulin resistance in a cross-sectional study on Chinese vegetarians and matched omnivores. We hypothesized that Se levels might improve insulin resistance in vegetarian**s.** To our knowledge, this is the first study to investigate the relationship between Se and insulin resistance among Chinese vegetarians.

## Methods

### Population

Vegetarians were recruited from Shanghai, China through vegetarian restaurants, vegetarian social activities, and online advertisements between the period of March 2016 and May 2016. The inclusion criteria were as follows: the participants had to 1) be at least 18 years of age; 2) reside in Shanghai at least for more than a period of 6 months; 3) follow a vegetarian diet for at least a year; 4) understand the contents of questionnaires. Exclusion criteria were based on 1) history of severe nutritional malabsorption or systemic diseases; 2) history of pregnancy or breastfeeding within the preceding 12 months. Omnivores were recruited within friends and relatives of the vegetarians and were matched respectively for the same sex and age (±1 year) to that of vegetarian participants. This study was approved by the Institutional Review Board of the Shanghai Jiao Tong University School of Medicine, and all participants provided written informed consent.

### Anthropometric indexes, clinical and dietary assessment

All subjects were questioned about their demographics and personal behavior information, including age, sex, income, alcohol consumption, smoking, physical activity, sedentary time, sleep duration, vegetarian pattern (vegan or lacto-ovo-vegetarian), and vegetarian duration, with a questionnaire. Vegetarians were defined as people who followed a vegetarian diet at all daily meals for at least 1 year; otherwise, they were defined as omnivores. Vegans were defined as those who did not consume any sort of foods of animal origin, while those who consumed meat, eggs, dairy products, or fish were defined as “lacto-ovo-vegetarians”.

Participants’ height, body weight, waist circumference, and hip circumference were obtained from the physical examination by trained dietitians following the standardized protocol. Body mass index (BMI) was calculated as weight (kilograms) divided by height (meters) squared. Waist-to-hip ratio was calculated as the measured waist circumference divided by hip circumference.

A 24-h diet recall questionnaire was used to assess their daily consumption of different nutrients. Daily nutrient intakes were calculated from the 24-h dietary recall data using Nutrition Calculator v2.5 software, which was developed by the Institute for Nutrition and Food Safety of the Chinese Centre for Disease Control and Prevention and Beijing B-win Technology Co. Ltd.

### Assessment of toenail Se exposure

282 vegetarians and 282 matched omnivores were recruited in the study. Participants were asked to provide toenail clippings from all ten toes and return them to the researchers. Sixty-two subjects did not provide their toenail samples. In this study, toenail samples were obtained from 220 vegetarians and 220 matched omnivores who met the criteria for inclusion. All toenail samples were pretreated by graphite digestion. The concentrations of toenail Se were measured according to inductively coupled plasma-mass spectrometry from the National Standard for Food Safety Determination of Multi Elements in Food (GB 5009.268–2016). Briefly, toenail samples (0.1 g) were placed in 50 ml PP centrifuge tube. After adding 0.5 ml HNO_3_ solution (Guaranteed reagent, Merck, Germany) and 0.5 ml H_2_O_2_ solution (Guaranteed reagent, LookChem, China), the mixtures were put in a graphite digester for digestion at 120 °C for 2 h. After digestion, the mixtures were taken out and cooled to room temperature. An inductively coupled plasma mass spectrometer (ICP-MS) ThermoFisher i CAP Q (ThermoFisher, USA) was used to analyze the levels of toenail Se. The lower detection limit and upper detection limit of Se in nails by ICP-MS, were 0.003 μg/g and 3.2 μg/g, respectively. The detection of Se level was conducted by professional researchers from Shanghai Jiao Tong University School of Agriculture and Biology.

### Assessment of insulin resistance

Peripheral venous blood samples were collected after at least 12 h of fasting. Fasting blood glucose (FG) and fasting insulin (FI) concentrations were tested by the Clinical Laboratory Center of Shanghai Xinhua Hospital. Insulin resistance and b-cell function were evaluated by the homeostasis model assessment (HOMA) method [18], in which FI (mU/L) and FG (mmol/L) were used.

HOMA-IR = FI (mU/L) × FG (mmol/L) / 22.5 [16, 19]

Insulin resistance is defined as HOMA-IR > 2.60 [18, 20].

HOMA-B(%) = 20 × FI (mU/L) / [FG (mmol/L)- 3.5] [16, 19]

### Statistical analysis

All statistical analyses were performed with the use of Stata software version 14.0 (StataCorp, College Station, TX, USA), and two-sided *P* values < 0.05 were considered as statistically significant. The continuous variables were shown as mean ± standard deviation (SD), while the categorical variables were expressed as number and percentage (%). To compare the differences between vegetarian group and omnivore group, the paired Student’s t-test study for continuous variables and paired chi-square test for categorical variables were used. Covariance analysis was used to test the differences in glucose metabolism and insulin resistance between the vegan group and the lacto-ovo-vegetarian group after controlling the covariates, including age, sex, BMI, alcohol consumption, income, and daily dietary intakes (energy, protein, fat, carbohydrate, and fiber). Then multi-linear regression was used to investigate the association between nail concentrations of Se and insulin resistance after adjusting for all major confounders. Interaction and stratified analyses were conducted according to sex (male and female) and vegetarian type (Vegan and Lacto-ovo-vegetarian).

## Results

The characteristics of the participants are shown in Table 1. In total, 220 vegetarians and 220 matched omnivores met the inclusion criteria were included in this study. The mean ages of vegetarians and omnivores were 35.96 ± 8.73 years and 35.23 ± 8.93 years, respectively. In the vegetarian group, 76 of the vegetarians were vegans, 144 were lacto-ovo vegetarians, and they had adhered to a vegetarian diet for more than 5 years. Of these, 180 (81.8%) were female and 40 (18.2%) were male. Vegetarians had a lower BMI, waist-to-hip ratio values, alcohol consumption, daily intakes of energy, protein, and fat (all *P* < 0·05), but higher income, physical activity, and daily intakes of fiber (all *P* < 0·05), compared with omnivores. In addition, the level of FG, FI, HOMA-IR, and dietary Se intake were significantly lower in vegetarians than in omnivores (all *P* < 0·05), while the number of participants under recommended nutrient intake Se level were higher in vegetarians than in omnivores (*P* < 0·05). The proportion of participants with insulin resistance was also lower in vegetarians at 1.36% than in omnivores at 5.45% (*P* < 0.05). The mean concentration of selenium in toenails of vegetarians was 0.53 ± 0.16 μg/g, which was significantly lower than that of omnivores (0.69 ± 0.53 μg/g, *P* < 0.05). There was also a significant difference in toenail Se between the vegan group (0.46 ± 0.11 μg/g) and the lacto-ovo-vegetarian group (0.56 ± 0.17 μg/g).

**Table 1:** Characteristics of the study population

	Vegan (*n* = 76)	Lacto-ovo-vegetarian (*n* = 144)	Vegetarian (*n* = 220)	Omnivore (*n* = 220)
Age (y)	36.84 ± 8.51	35.49 ± 8.85	35.96 ± 8.73	35.23 ± 8.93
Female, %	77.63	84.03	81.82	81.82
Vegetarian duration (y)	5.79 ± 4.46	5.52 ± 5.33	5.61 ± 5.04	–
BMI (kg/m^2^)	20.72 ± 2.27	21.17 ± 2.67	21.02 ± 2.54*	22.53 ± 3.48
Waist-to-hip ratio	0.81 ± 0.05	0.81 ± 0.05	0.81 ± 0.05*	0 .84 ± 0 .05
No alcohol, %	98.68	93.75	95.45*	81.36
No smoking, %	86.84	90.97	89.55	90.45
Physical activity (min/wk)	151.97 ± 181.98	93.16 ± 108.48	113.48 ± 140.75*	82.86 ± 114.39
Income (Yuan/month), %				
< 3000	17.11	19.44	18.64*	25.45
3000 ~ 5000	22.37	17.36	19.09*	20
5000 ~ 8000	18.42	29.17	25.45*	24.10
> 8000	42.10	34.03	36.82*	30.45
Energy (Kcal/d)	1536.32 ± 541.52	1496.98 ± 503.67	1510.57 ± 516.17*	1792.88 ± 583.46
Protein (g/d)	48.50 ± 23.94	44.48 ± 18.92	45.87 ± 20.83*	71.52 ± 31.64
Fat (g/d)	39.49 ± 21.22	43.02 ± 21.31	41.80 ± 21.30*	68.14 ± 34.66
Carbohydrate (g/d)	237.96 ± 90.39	227.55 ± 84.11	231.14 ± 86.26	217.14 ± 75.72
Fiber (g/d)	19.36 ± 11.89	13.63 ± 7.56**	15.61 ± 9.65*	12.04 ± 7.12
FG (mmol/L)	4.58 ± 0.29	4.66 ± 0 .80	4.63 ± 0.67*	4.82 ± 0.40
FI (mU/L)	4.76 ± 2.67	4.96 ± 2.08	4.89 ± 2.30*	6.03 ± 3.11
HOMA-IR	0.98 ± 0.56	1.05 ± 0.60	1.03 ± 0.59*^(1)^	1.30 ± 0.71
HOMA-β (%)	91.26 ± 58.47	94.85 ± 42.97	93.61 ± 48.78	97.86 ± 62.42
Insulin resistance (%)	1.32	1.39	1.36%*	5.45
Dietary Se intake (μg/d)	25.28 ± 23.89	25.83 ± 15.03	25.64 ± 18.52*	55.14 ± 37.51
Toenail Se (μg/g)	0.46 ± 0.11	0.56 ± 0.17**	0.53 ± 0.16*^(1)^	0.69 ± 0.53

*Abbreviations*: *BMI* body mass index, *FG* fasting blood glucose, *FI* fasting insulin, *HOMA-IR* homeostasis model assessment of insulin resistance, *HOMA-β* homeostasis model assessment of β cell function

^(1)^Data were assessed with covariance controlling for age, sex, BMI, alcohol consumption, income, and daily dietary intakes (energy, protein, fat, carbohydrate, and fiber)

*Statistical significance when comparing vegetarian and omnivore groups

**Statistical significance when comparing vegan and lacto-ovo-vegetarian groups

The multiple regression analysis for associations between toenail Se levels and glucose metabolic indexes is presented in Table 2. The multi-linear regression results showed that omnivore diet was positively associated with FI (*β* = 1.030, 95%CI: 0.393 to 1.667) and HOMA-IR (*β* = 0.245, 95%CI: 0.098 to 0.392) after adjusting for age, sex, BMI, alcohol consumption, income, and daily dietary intakes (energy, protein, fat, carbohydrate, and fiber). No significant differences were found between vegetarian diet and FI as well as HOMA-IR. After adjustment for confounders, no significant interactions were observed between toenail Se and glucose metabolic indexes (FG, FI, HOMA-IR, and HOMA-β) according to sex and vegetarian type among omnivores (Table S1) and vegetarians (Table S2). Similarly, the differences between dietary Se intake and glucose metabolic indexes were not statistically significant between men and women (Table S3 and S4) or between vegans and lacto-ovo-vegetarians (Table S4). When we examined nail Se concentration as a tertile variable, no significant difference was seen between nail Se tertiles and glucose metabolic indexes (FG, FI, HOMA-IR, and HOMA-β) among omnivores (Table S5) and vegetarians (Table S6) after adjusting for confounders.

**Table 2:** Multiple regression analysis for associations between toenail Se levels and glucose metabolic indexes in vegetarians and omnivores

		Vegetarian (*n* = 220)		Omnivore (*n* = 220)	
		*β* (95%CI)	*P*	*β* (95%CI)	*P*
FG (mmol/L)	Model 1	0.340 (− 0.224, 0.905)	0.24	0.079 (− 0.022, 0.180)	0.13
	Model 2	0.303 (− 0.278, 0.885)	0.31	0.029 (− 0.065, 0.122)	0.55
FI	Model 1	1.427 (−0.503, 3.357)	0.15	1.294 (0.532, 2.056)	< 0.01
	Model 2	1.190 (−0.707, 3.086)	0.22	1.030 (0.393, 1.667)	< 0.01
HOMA-IR	Model 1	0.358 (−0.136, 0.851)	0.16	0.319 (0.144, 0.493)	< 0.01
	Model 2	0.288 (−0.201, 0.776)	0.25	0.245 (0.098, 0.392)	< 0.01
HOMA-B(%)	Model 1	13.560 (−27.543, 54.663)	0.52	8.99 (−6.67, 24.65)	0.26
	Model 2	8.568 (−32.381, 49.517)	0.68	9.99 (−4.44, 24.43)	0.18

*Abbreviations*: *FG* fasting blood glucose, *FI* fasting insulin, *HOMA-IR* homeostasis model assessment of insulin resistance, *HOMA-β* homeostasis model assessment of β cell function

Model 1:unadjusted regression

Model 2:regression with age, sex, BMI, alcohol consumption, income, and daily dietary intakes (energy, protein, fat, carbohydrate, and fiber) controlled

The multiple regression analysis for associations between toenail Se levels and glucose metabolic indexes according to dietary Se intake in omnivores is displayed in Table 3. The concentration of Se in toenails was positively associated with FI (*β* = 1.053, 95%*CI*: 0.415 to 1.691), HOMA-IR (*β* = 0.237, 95%*CI*: 0.079 to 0.395), and HOMA-β (*β* = 13.271, 95%*CI*: 4.433 to 22.109) in omnivores with dietary intake of Se above 60 μg/d (China recommended nutrient intake (RNI) level), however, no association was observed, when the dietary Se intake was below 60 μg/d, after multivariate adjustment for age, sex, BMI, alcohol consumption, income, and daily dietary intakes (energy, protein, fat, carbohydrate, and fiber).

**Table 3:** Multiple regression analysis for associations between toenail Se levels and glucose metabolic indexes according to dietary Se intake in omnivores

Dietary Se intake (μg/d)	< 60 (*n* = 155)			≥60 (*n* = 65)	
		*β* (95%CI)	*P*	*β* (95%CI)	*P*
FG (mmol/L)	Model 1	0.391 (0.059, 0.723)	0.06	0.034 (−0.072, 0.140)	0.53
	Model 2	0.166 (−0.159, 0.491	0.32	−0.025 (− 0.123, 0.073)	0.62
FI (mU/L)	Model 1	2.330 (−0.410, 5.070)	0.10	1.161 (0.469, 1.853)	< 0.01
	Model 2	2.146 (−0.710, 5.002)	0.14	1.053 (0.415, 1.691)	< 0.01
HOMA-IR	Model 1	0.614 (0.001, 1.227	0.05	0.279 (0.112, 0.445)	< 0.01
	Model 2	0.504 (−0.140, 1.148)	0.13	0.237 (0.079, 0.395)	< 0.01
HOMA-B (%)	Model 1	−4.121 (−63.969, 55.726)	0.89	11.041 (0.670, 21.412)	< 0.01
	Model 2	13.766 (−46.868, 74.400)	0.66	13.271 (4.433, 22.109)	< 0.01

*Abbreviations*: *FG* fasting blood glucose, *FI* fasting insulin, *HOMA-IR* homeostasis model assessment of insulin resistance, *HOMA-β* homeostasis model assessment of β cell function

Model 1:unadjusted regression

Model 2:regression with age, sex, BMI, alcohol consumption, income, and daily dietary intakes (energy, protein, fat, carbohydrate, and fiber) controlled

## Discussion

In this study, no association was observed between toenail Se levels and insulin resistance in vegetarians, however, higher toenail Se levels elevated the risk of insulin resistance in omnivores, after adjusting all major confounding factors. Moreover, this phenomenon persisted in omnivores whose dietary Se intake exceeded 60 μg/d, but not in those with levels below 60 μg/d.

To the best of our knowledge, this was the first study to investigate the relationship between Se and insulin resistance among Chinese vegetarians. Our findings are congruent to those of a recent cross-sectional study which found that higher nail Se levels were associated with upregulated HOMA-IR in older people in rural China [17]. The adjusted β value for the highest Se quartile group (≥0.568 μg/g) was 0.34 (95% CI: 0.08 to 0.80), compared with the first Se quartile group (< 0.320 μg/g) [17]. Similar results were reported in another study in which a positive relationship was found between serum Se levels and HOMA-IR in aged Polish men with metabolic syndrome [21]. Unlike previous studies that focused on older participants [17, 21], this study provides important insights into the association between toenail Se levels and insulin resistance in younger populations. Our findings agree with previous reports that higher level of toenail Se might be associated with the higher risk of diabetes [11, 17].

However, our results differ from those of previous studies in which higher levels of toenail Se were found to decrease the risk of diabetes in men and women in the U.S. [13]. The ORDET cohort study reported that toenail Se was not associated with the incidence of diabetes [14]. These inconsistent results may be explained by the different outcomes measures across studies. The outcome of our study was insulin resistance, while studies with contradictory findings assessed the incidence of diabetes as the target outcome. Insulin resistance has been identified as one of risk factors for the development of diabetes [22, 23]. Given that high physical activity and weight loss decrease insulin resistance, these two factors may reduce incidence of diabetes [23], and lead to inconsistent conclusions from different studies. The discrepant results may be due to differences in the distribution of confounders for related insulin resistance and diabetes, such as lifestyle factors and genetic susceptibility to insulin resistance and diabetes [24].

As expected, we found a positive correlation between dietary Se intake and toenail Se level among the subjects in Table S7 (*r* = 0.174, *P* < 0.01), after adjusting major confounding factors. This implies that dietary Se intake affects the toenail Se level, while the toenail Se level reflects dietary Se intake. Taking into account the practical guidance on dietary Se intake, omnivores were divided into two groups using 60 μg/d (China RNI level) as the reference value in Table 3. Notably, when dietary Se intake exceeded 60 μg/d, higher toenail Se level correlated positively with FI and HOMA-IR. However, no significant association was observed in subjects when the dietary intake of Se was under 60 μg/d. Previous studies have suggested that high levels of Se may be associated with insulin resistance and higher risk of diabetes [24]. Our finding is in agreement with that of the ORDET cohort study in which the odds ratio for diabetes comparing the highest (55 μg/d) and the lowest (32 μg/d) quintile of Se intake was 1.74, (95% CI: 1.12, 2.72; P for linear trend 0.001), after adjusting for age, education and menopausal status [12]. These findings create awareness on t the association of dietary Se intake above 60 μg/d with risk of insulin resistance in Chinese omnivores. Further studies with larger sample sizes from different populations are required to explore the safety range of dietary Se intake.

In the present study, no statistically significant differences between toenail Se levels and insulin resistance were found in the vegetarian group. This result may partly be explained by the low dietary Se intake in vegetarians. The mean dietary Se intake of vegetarians was 25.64 ± 18.52 μg/d, which was significantly lower than that of omnivores (55.14 ± 37.51, *P* < 0.05). Previous studies have observed a trend of U-shaped relationship between serum Se level and the risk of diabetes [25]. Therefore, when the Se levels were relatively low, the association between Se levels and insulin resistance may not be observed. Another possible explanation for this finding is that the vegetarian diet may have a potential protective effect on the improvement of insulin resistance. A plant-based diet with various foods rich in anti-oxidants and phytochemicals, which may have a direct effect on alleviating oxidative stress and inflammation, may account for the lower insulin resistance among vegetarians [26]. A recent prospective study indicated that higher consumptions of phytochemical-rich foods may improve the development of IR [26]. Hence, the effect of Se on insulin resistance was reduced because of the protective effect of a vegetarian diet. Future studies are needed to investigate the potential underlying mechanisms.

Several mechanisms may explain how Se affects insulin resistance. Some of such mechanisms include insulin-like action, oxidative stress, and inflammatory cytokines [16]. In animal studies, diets rich in Se may promote the release of glucagon causing s hyperglycemia. Additionally, diets rich in Se may increase the expression of glutathione peroxidase-1 and other anti-oxidant selenoproteins leading to insulin resistance and obesity [12]. Previously, a positive association between glutathione peroxidase activity and insulin resistance was observed in non-diabetic women during normal pregnancy [27]. Mechanistically, dietary Se intake above the recommended level, for optimal activity of antioxidant selenoproteins like glutathione peroxidases (55 μg/d), will lead to non-specific incorporation of selenomethionine thereby replacing methionine in albumin and other proteins [28, 29].

One of the strengths of this study is that Se was measured via toenail samples, which reflected a relatively long-term measure of Se exposure, in comparison with serum or urine samples. In addition, the Se levels in toenail did not fluctuate remarkably with the daily dietary Se intake [30]. Secondly, many major confounding factors were controlled, ensuring the findings in this study to be more accurate. Thirdly, dietary assessments were conducted by trained and professional Chinese registered dietitians, and strict quality control measures were adopted throughout the study.

Some potential limitations in the present study need to be acknowledged. Firstly, our study sample was relatively small, so larger studies are required to be conducted in the future. Secondly, the findings were limited by the use of a cross-sectional design. Thirdly, this study did not consider genetic factors or other environmental factors that might influence the association between Se levels and the risk of insulin resistance. Therefore, more researches need to be undertaken before the association between Se and insulin resistance is more clearly understood.

## Conclusions

In conclusion, our findings reveal a significantly positive association of toenail Se levels with insulin resistance in Chinese omnivores after adjusting major confounding factors. This positive relationship persists in omnivores whose dietary Se intake exceeds 60 μg/d, but not in those with levels below 60 μg/d. These results create awareness on the association of dietary Se intake above 60 μg/d with the risk of insulin resistance.

This present study adds to the evidence of the association between high toenail Se levels and potential insulin resistance risk. Further research should be undertaken to investigate the association between Se exposure and metabolic effects, as well as potential underlying mechanisms.

## Supplementary information

**Additional file 1: Table S1.** Multiple regression analysis for associations between toenail Se levels and glucose metabolic indexes according to sex in omnivores. **Table S2.** Multiple regression analysis for associations between toenail Se levels and glucose metabolic indexes according to sex and vegetarian type in vegetarians. **Table S3.** Multiple regression analysis for associations between dietary selenium intake and glucose metabolic indexes in omnivores. **Table S4.** Multiple regression analysis for associations between dietary selenium intake and glucose metabolic indexes in vegetarians. **Table S5.** β value and 95% confidence interval for glucose metabolic indexes according to tertiles of nail selenium in omnivores. **Table S6.** β value and 95% confidence interval for glucose metabolic indexes according to tertiles of nail selenium in vegetarians. **Table S7.** Pearson correlation analysis between dietary Se intake and toenail Se level.

## Data Availability

The datasets used and/or analysed during the current study are available from the corresponding author on reasonable request.

## Abbreviations

BMI: Body mass index
FG: Fasting blood glucose
FI: Fasting insulin
HOMA-IR: Homeostasis model assessment of insulin resistance
HOMA-β: Homeostasis model assessment of β cell function
T2DM: Type 2 diabetes mellitus
Se: Selenium
