# Supplementary material for: Higher toenail selenium is associated with increased insulin resistance risk in omnivores, but not in vegetarians
Source: Nutr Metab (Lond). 2020 Aug 3;17:62. doi: 10.1186/s12986-020-00484-6 (PMC7398369; doi:10.1186/s12986-020-00484-6)
Supplement: Supplementary file 1 — Additional file 1: Table S1. Multiple regression analysis for associations between toenail Se levels and glucose metabolic indexes according to sex in omnivores. Table S2. Multiple regression analysis for associations between toenail Se levels and glucose metabolic indexes according to sex and vegetarian type in vegetarians. Table S3. Multiple regression analysis for associations between dietary selenium intake and glucose metabolic indexes in omnivores. Table S4. Multiple regression analysis for associations between dietary selenium intake and glucose metabolic indexes in vegetarians. Table S5. β value and 95% confidence interval for glucose metabolic indexes according to tertiles of nail selenium in omnivores. Table S6. β value and 95% confidence interval for glucose metabolic indexes according to tertiles of nail selenium in vegetarians. Table S7. Pearson correlation analysis between dietary Se intake and toenail Se level. [file 12986_2020_484_MOESM1_ESM.docx]

**Table S1 Multiple regression analysis for associations between toenail Se levels and glucose metabolic indexes according to sex in omnivores.**

|  | **N** | **Model 1** |  | ***P* value for interaction** | **Model 2** |  | ***P* value for interaction** |
| --- | --- | --- | --- | --- | --- | --- | --- |
|  |  | **β (95%CI)** | ***P*** |  | **β (95%CI)** | ***P*** |  |
| **FG (mmol/L)** |  |  |  | 0.14 |  |  | 0.54 |
| Male | 40 | 0.049 (-0.058, 0.156) | 0.38 |  | 0.007 (-0.097, 0.112) | 0.89 |  |
| Female | 180 | 0.325 (-0.029, 0.678) | 0.07 |  | 0.144 (-0.188, 0.477) | 0.40 |  |
| **FI (mU/L)** |  |  |  | 0.76 |  |  | 0.49 |
| Male | 40 | 1.243 (0.487, 1.999) | <0.01 |  | 1.083 (0.411, 1.756) | <0.01 |  |
| Female | 180 | 1.680 (-1.056, 4.416) | 0.23 |  | -0.049 (-2.390, 2.292) | 0.97 |  |
| **HOMA-IR** |  |  |  |  |  |  | 0.54 |
| Male | 40 | 0.299 (0.122, 0.476) | <0.01 | 0.62 | 0.253 (0.095, 0.411) | <0.01 |  |
| Female | 180 | 0.462 (-0.159, 1.083) | 0.14 |  | 0.024 (-0.518, 0.565) | 0.93 |  |
| **HOMA-β (%)** |  |  |  |  |  |  | 0.32 |
| Male | 40 | 11.69 (1.12, 22.27) | <0.05 | 0.39 | 12.84 (2.97, 22.71) | <0.05 |  |
| Female | 180 | -13.17 (-71.72, 45.39) | 0.66 |  | -21.63 (-76.35, 33.09) | 0.44 |  |

**Model 1, unadjusted regression;**

**Model 2：regression with age, BMI, alcohol consumption, income, and daily dietary intakes (energy, protein, fat, carbohydrate, and fiber) controlled;**

**Table S2 Multiple regression analysis for associations between toenail Se levels and glucose metabolic indexes according to sex and vegetarian type in vegetarians**

|  | **N** | **Model 1** |  | ***P* value for interaction** | **Model 2** |  | ***P* value for interaction** |
| --- | --- | --- | --- | --- | --- | --- | --- |
|  |  | **β (95%CI)** | ***P*** |  | **β (95%CI)** | ***P*** |  |
| **FG (mmol/L)** | 220 |  |  |  |  |  |  |
| Sex |  |  |  | 0.61 |  |  | 0.87 |
| Male | 40 | 0.601 (-0.045, 1.246) | 0.08 |  | 0.647 (-0.167, 1.461) | 0.13 |  |
| Female | 180 | 0.260 (-0.435, 0.956) | 0.46 |  | 0.338 (-0.389, 1.064) | 0.36 |  |
| Vegetarian type |  |  |  | 0.56 |  |  |  |
| Vegan | 76 | -0.063 (-0.653, 0.527) | 0.83 |  | 0.033 (-0.574, 0.641) | 0.91 |  |
| Lacto-Ovo | 144 | 0.384 (-0.404, 1.172) | 0.34 |  | 0.336 (-0.491, 1.164) | 0.43 |  |
| **FI (mU/L)** | 220 |  |  |  |  |  |  |
| Sex |  |  |  | 0.49 |  |  | 0.30 |
| Male | 40 | 2.618 (-1.715, 6.950) | 0.24 |  | -1.215 (-6.453, 4.024) | 0.65 |  |
| Female | 180 | 1.033 (-1.139, 3.205) | 0.35 |  | 0.469 (-1.656, 2.594) | 0.67 |  |
| Vegetarian type |  |  |  | <0.05 |  |  | 0.06 |
| Vegan | 76 | 6.358 (1.112, 11.603) | <0.05 |  | 5.166 (-0.264, 10.595) | 0.07 |  |
| Lacto-Ovo | 144 | 0.193 (-1.858, 2.244) | 0.85 |  | 0.164 (-1.927, 2.254) | 0.88 |  |
| **HOMA-IR** | 220 |  |  |  |  |  |  |
| Sex |  |  |  | 0.50 |  |  | 0.53 |
| Male | 40 | 0.660 (-0.307, 1.627) | 0.19 |  | -0.094 (-1.248, 1.060) | 0.87 |  |
| Female | 180 | 0.259 (-0.313, 0.832) | 0.38 |  | 0.181 (-0.396, 0.757) | 0.54 |  |
| Vegetarian type |  |  |  | 0.10 |  |  | 0.18 |
| Vegan | 76 | 1.196 (0.093, 2.299) | <0.05 |  | 0.987 (-0.121, 2.096) | 0.09 |  |
| Lacto-Ovo | 144 | 0.113 (-0.480, 0.707) | 0.71 |  | 0.076 (-0.533, 0.685) | 0.81 |  |
| **HOMA-β (%)** | 220 |  |  |  |  |  |  |
| Sex |  |  |  | 0.94 |  |  | 0.64 |
| Male | 40 | 10.07 (-64.10, 84.25) | 0.79 |  | -75.157 (-170.88, 20.57) | 0.14 |  |
| Female | 180 | 13.57 (-34.72, 61.87) | 0.58 |  | 2.456 (-45.125, 50.037) | 0.92 |  |
| Vegetarian type |  |  |  | <0.01 |  |  | 0.06 |
| Vegan | 76 | 174.21 (61.8, 286.7) | <0.01 |  | 136.41 (8.37, 264.45) | 0.06 |  |
| Lacto-Ovo | 144 | -26.91 (-68.99, 15.16) | 0.21 |  | -28.413 (-70.458, 13.633) | 0.19 |  |

**Model 1, unadjusted regression;**

**Model 2：regression with age, BMI, alcohol consumption, income, and daily dietary intakes (energy, protein, fat, carbohydrate, and fiber) controlled;**

**Table S3 Multiple regression analysis for associations between dietary selenium intake and glucose metabolic indexes in omnivores**

|  | **N** | **Model 1** |  | ***P* value for interaction** | **Model 2** |  | ***P* value for interaction** |
| --- | --- | --- | --- | --- | --- | --- | --- |
|  |  | **β (95%CI)** | ***P*** |  | **β (95%CI)** | ***P*** |  |
| **FG (mmol/L)** |  |  |  |  |  |  |  |
| All | 220 | 0.002 (0.000, 0.003) | <0.05 |  | 0.002 (-0.000, 0.004) | 0.11 |  |
| Male | 40 | 0.003 (-0.001, 0.007) | 0.11 | 0.30 | 0.005 (-0.000, 0.011) | 0.07 | 0.14 |
| Female | 180 | 0.001 (-0.000, 0.003) | 0.09 |  | 0.001 (-0.001, 0.003) | 0.37 |  |
| **FI (mU/L)** |  |  |  |  |  |  |  |
| All | 220 | 0.008 (-0.003, 0.019) | 0.15 |  | 0.004 (-0.010, 0.019) | 0.56 |  |
| Male | 40 | 0.027 (-0.001, 0.054) | 0.07 | 0.07 | 0.016 (-0.018, 0.051) | 0.36 | 0.18 |
| Female | 180 | 0.004 (-0.008, 0.016) | 0.52 |  | -0.000 (-0.017, 0.016) | 0.96 |  |
| **HOMA-IR** |  |  |  |  |  |  |  |
| All | 220 | 0.002 (-0.000, 0.005) | 0.09 |  | 0.002 (-0.002, 0.005) | 0.37 |  |
| Male | 40 | 0.007 (0.000, 0.013) | <0.05 | <0.05 | 0.005 (-0.002, 0.013) | 0.19 | 0.10 |
| Female | 180 | 0.001 (-0.002, 0.004) | 0.40 |  | 0.000 (-0.003, 0.004) | 0.89 |  |
| **HOMA-β (%)** |  |  |  |  |  |  |  |
| All | 220 | -0.066 (-0.287, 0.155) | 0.56 |  | -0.111 (-0.428, 0.205) | 0.49 |  |
| Male | 40 | 0.174 (-0.229, 0.577) | 0.40 | 0.28 | -0.221 (-0.783, 0.341) | 0.45 | 0.99 |
| Female | 180 | -0.109 (-0.360, 0.142) | 0.40 |  | -0.100 (-0.476, 0.275) | 0.60 |  |

**Model 1, unadjusted regression;**

**Model 2：regression with age, BMI, alcohol consumption, income, and daily dietary intakes (energy, protein, fat, carbohydrate, and fiber) controlled;**

**Table S4 Multiple regression analysis for associations between dietary selenium intake and glucose metabolic indexes in vegetarians**

|  | **N** | **Model 1** |  | ***P* value for interaction** | **Model 2** |  | ***P* value for interaction** |
| --- | --- | --- | --- | --- | --- | --- | --- |
|  |  | **β (95%CI)** | ***P*** |  | **β (95%CI)** | ***P*** |  |
| **FG (mmol/L)** |  |  |  |  |  |  |  |
| All | 220 | 0.001 (-0.004, 0.006) | 0.65 |  | 0.000 (-0.005, 0.006) | 0.89 |  |
| Sex |  |  |  | 0.89 |  |  | 0.99 |
| Male | 40 | -0.000 (-0.013, 0.012) | 0.96 |  | 0.006 (-0.011, 0.022) | 0.52 |  |
| Female | 180 | 0.001 (-0.004, 0.006) | 0.66 |  | 0.000 (-0.006, 0.007) | 0.89 |  |
| Vegetarian type |  |  |  | 0.52 |  |  | 0.90 |
| Vegan | 76 | -0.000 (-0.003, 0.002) | 0.84 |  | 0.001 (-0.003, 0.004) | 0.64 |  |
| Lacto-Ovo | 144 | 0.003 (-0.006, 0.012) | 0.52 |  | 0.002 (-0.010, 0.014) | 0.76 |  |
| **FI (mU/L)** |  |  |  |  |  |  |  |
| All | 220 | -0.002 (-0.019, 0.014) | 0.81 |  | -0.009 (-0.027, 0.010) | 0.34 |  |
| Sex |  |  |  | 0.17 |  |  | 0.12 |
| Male | 40 | -0.051 (-0.131, 0.028) | 0.21 |  | 0.017 (-0.081, 0.114) | 0.74 |  |
| Female | 180 | 0.001 (-0.016, 0.018) | 0.92 |  | -0.002 (-0.020, 0.016) | 0.84 |  |
| Vegetarian type |  |  |  | 0.63 |  |  | 0.90 |
| Vegan | 76 | -0.006 (-0.031, 0.020) | 0.67 |  | -0.018 (-0.051, 0.014) | 0.27 |  |
| Lacto-Ovo | 144 | 0.002 (-0.020, 0.025) | 0.83 |  | -0.015 (-0.046, 0.016) | 0.35 |  |
| **HOMA-IR** |  |  |  |  |  |  |  |
| All | 220 | 0.000 (-0.004, 0.004) | 0.97 |  | -0.001 (-0.006, 0.003) | 0.60 |  |
| Sex |  |  |  | 0.22 |  |  | 0.21 |
| Male | 40 | -0.011 (-0.029, 0.007) | 0.23 |  | 0.004 (-0.017, 0.026) | 0.69 |  |
| Female | 180 | 0.001 (-0.004, 0.005) | 0.74 |  | 0.000 (-0.005, 0.005) | 0.94 |  |
| Vegetarian type |  |  |  | 0.53 |  |  | 0.95 |
| Vegan | 76 | -0.001 (-0.006, 0.004) | 0.67 |  | -0.003 (-0.010, 0.003) | 0.31 |  |
| Lacto-Ovo | 144 | 0.002 (-0.005, 0.008) | 0.64 |  | -0.002 (-0.011, 0.007) | 0.67 |  |
| **HOMA-β (%)** |  |  |  |  |  |  |  |
| All | 220 | -0.102 (-0.452, 0.247) | 0.57 |  | -0.194 (-0.586, 0.198) | 0.33 |  |
| Sex |  |  |  | 0.33 |  |  | 0.30 |
| Male | 40 | -0.827 (-2.163, 0.508) | 0.23 |  | -0.279 (-2.190, 1.632) | 0.78 |  |
| Female | 180 | -0.051 (-0.425, 0.324) | 0.79 |  | -0.109 (-0.513, 0.295) | 0.60 |  |
| Vegetarian type |  |  |  | 0.88 |  |  | 0.63 |
| Vegan | 76 | -0.127 (-0.684, 0.430) | 0.66 |  | -0.500 (-1.263, 0.262) | 0.20 |  |
| Lacto-Ovo | 144 | -0.072 (-0.542, 0.398) | 0.76 |  | -0.467 (-1.095, 0.160) | 0.15 |  |

**Model 1, unadjusted regression;**

**Model 2：regression with age, BMI, alcohol consumption, income, and daily dietary intakes (energy, protein, fat, carbohydrate, and fiber) controlled;**

**Table S5 β value and 95% confidence interval for glucose metabolic indexes according to tertiles of nail selenium in omnivores**

|  | Toenail Se (μg/g) | N | Model 1 |  | Model 2 |  |
| --- | --- | --- | --- | --- | --- | --- |
|  |  |  | β (95%CI) | *P* | β (95%CI) | *P* |
| FG (mmol/L) | T_1_ | 73 | 0.00 (reference) |  | 0.00 (reference) |  |
|  | T_2_ | 73 | -0.022 (-0.151,0.107) | 0.74 | 0.002 (-0.120, 0.123) | 0.98 |
|  | T_3_ | 74 | 0.167 (0.038, 0.295) | <0.05 | 0.130 (0.010, 0.249) | 0.06 |
|  | *P* for trend |  |  | <0.05 |  | 0.06 |
| FI (mU/L) | T_1_ | 73 | 0.00 (reference) |  | 0.00 (reference) |  |
|  | T_2_ | 73 | -0.600 (-1.606, 0.405) | 0.24 | -0.471 (-1.332, 0.390) | 0.30 |
|  | T_3_ | 74 | 0.457 (-0.545, 1.458) | 0.37 | 0.48 (-0.368, 1.321) | 0.26 |
|  | *P* for trend |  |  | 0.37 |  | 0.37 |
| HOMA-IR | T_1_ | 73 | 0.00 (reference) |  | 0.00 (reference) |  |
|  | T_2_ | 73 | -0.116 (-0.346, 0.114) | 0.33 | -0.079 (-0.278, 0.119) | 0.44 |
|  | T_3_ | 74 | 0.15 (-0.079, 0.379) | 0.20 | 0.14 (-0.056, 0.333) | 0.16 |
|  | *P* for trend |  |  | 0.20 |  | 0.22 |
| HOMA-β (%) | T_1_ | 73 | 0.00 (reference) |  | 0.00 (reference) |  |
|  | T_2_ | 73 | -16.899 (-37.108, 3.310) | 0.10 | -18.147 (-36.401, 1.834) | 0.08 |
|  | T_3_ | 74 | -12.150 (-32.291, 7.991) | 0.24 | -7.698 (-26.383, 10.987) | 0.45 |
|  | *P* for trend |  |  | 0.24 |  | 0.27 |

**Model 1, unadjusted regression;**

**Model 2：regression with age, sex, BMI, alcohol consumption, income, and daily dietary intakes (energy, protein, fat, carbohydrate, and fiber) controlled;**

**Abbreviations: FG, fasting blood glucose; FI, fasting insulin; HOMA-IR, homeostasis model assessment of insulin resistance; HOMA-β, homeostasis model assessment of β cell function; N, Number; T, tertile. The cutoff values for Se were 0.598μg/g and 0.696μg/g in omnivores. T_1_:(<0.598μg/g); T_2_:(0.598-0.696μg/g);T_3_:(≥0.696μg/g).**

**Table S6 β value and 95% confidence interval for glucose metabolic indexes according to tertiles of nail selenium in vegetarians**

|  | Toenail Se (μg/g) | N | Model 1 |  | Model 2 |  |
| --- | --- | --- | --- | --- | --- | --- |
|  |  |  | β (95%CI) | *P* | β (95%CI) | *P* |
| FG (mmol/L) | T_1_ | 73 | 0.00 (reference) |  | 0.00 (reference) |  |
|  | T_2_ | 73 | 0.108 (-0.099, 0.314) | 0.31 | 0.196 (-0.018, 0.409) | 0.07 |
|  | T_3_ | 74 | 0.092 (-0.147, 0.332) | 0.45 | 0.102 (-0.142, 0.346) | 0.41 |
|  | *P* for trend |  |  | 0.34 |  | 0.36 |
| FI (mU/L) | T_1_ | 73 | 0.00 (reference) |  | 0.00 (reference) |  |
|  | T_2_ | 73 | -0.004 (-0.711, 0.703) | 0.99 | 0.102 (-0.606, 0.811) | 0.78 |
|  | T_3_ | 74 | 0.363 (-0.457, 1.184) | 0.39 | 0.265 (-0.544, 1.075) | 0.52 |
|  | *P* for trend |  |  | 0.45 |  | 0.61 |
| HOMA-IR | T_1_ | 73 | 0.00 (reference) |  | 0.00 (reference) |  |
|  | T_2_ | 73 | 0.052 (-0.129, 0.233) | 0.57 | 0.107 (-0.075, 0.290) | 0.25 |
|  | T_3_ | 74 | 0.086 (-0.124, 0.296) | 0.42 | 0.069 (-0.139, 0.278) | 0.52 |
|  | *P* for trend |  |  | 0.39 |  | 0.55 |
| HOMA-β (%) | T_1_ | 73 | 0.00 (reference) |  | 0.00 (reference) |  |
|  | T_2_ | 73 | 4.351 (-10.669, 19.371) | 0.57 | 2.377 (-12.819, 17.574) | 0.76 |
|  | T_3_ | 74 | 2.283 (-15.155, 19.720) | 0.80 | -0.241 (-17.611, 17.129) | 0.98 |
|  | *P* for trend |  |  | 0.70 |  | 0.86 |

**Model 1, unadjusted regression;**

**Model 2：regression with age, sex, BMI, alcohol consumption, income, and daily dietary intakes (energy, protein, fat, carbohydrate, and fiber) controlled;**

**Abbreviations: FG, fasting blood glucose; FI, fasting insulin; HOMA-IR, homeostasis model assessment of insulin resistance; HOMA-β, homeostasis model assessment of β cell function; N, Number; T, tertile. The cutoff values for Se were 0.453μg/g and 0.557μg/g in vegetarians. T_1_:(<0.453μg/g); T_2_:(0.453-0.557μg/g);T_3_:(≥0.557μg/g).**

**Table S7 Pearson correlation analysis between dietary Se intake and toenail Se level**

|  | **Toenail Se level (N=440)** |  |
| --- | --- | --- |
|  | **r** | ***P*** |
| **Dietary Se intake (N=440)** | **0.174** | **<0.01** |
